# Regulation of Adaptive Immune Cells by Sirtuins

**DOI:** 10.3389/fendo.2019.00466

**Published:** 2019-07-11

**Authors:** Jonathan L. Warren, Nancie J. MacIver

**Affiliations:** ^1^Department of Pediatrics, Duke University School of Medicine, Durham, NC, United States; ^2^Department of Immunology, Duke University School of Medicine, Durham, NC, United States; ^3^Department of Pharmacology and Cancer Biology, Duke University School of Medicine, Durham, NC, United States

**Keywords:** adaptive immunity, T cells, epigenetics, sirtuins, metabolism

## Abstract

It is now well-established that the pathways that control lymphocyte metabolism and function are intimately linked, and changes in lymphocyte metabolism can influence and direct cellular function. Interestingly, a number of recent advances indicate that lymphocyte identity and metabolism is partially controlled via epigenetic regulation. Epigenetic mechanisms, such as changes in DNA methylation or histone acetylation, have been found to alter immune function and play a role in numerous chronic disease states. There are several enzymes that can mediate epigenetic changes; of particular interest are sirtuins, protein deacetylases that mediate adaptive responses to a variety of stresses (including calorie restriction and metabolic stress) and are now understood to play a significant role in immunity. This review will focus on recent advances in the understanding of how sirtuins affect the adaptive immune system. These pathways are of significant interest as therapeutic targets for the treatment of autoimmunity, cancer, and transplant tolerance.

## Introduction

The adaptive immune system is critical for responding to and eliminating foreign pathogens. T cells are important members of the adaptive immune system, and are generally responsible for recruiting additional inflammatory machinery to the site of infection or tumor. T cells develop within the thymus and, upon maturation, are classified broadly by their expression of either CD4 or CD8 receptor. Both CD4^+^ and CD8^+^ T cells exist as a number of subsets that perform unique functions within the immune milieu and exhibit unique surface receptors, produce lineage-specific cytokines, and express lineage-defining transcription factors. CD4^+^ T effector cells (Teff) are a broad class of T cells that are further divided into unique subsets with distinct effector functions. T helper 1 (Th1) cells are necessary for combating intracellular bacteria and viruses and for producing cytokines (most notably IFNγ and IL-2, but also TNFα) to promote cellular immunity, macrophage activation, and phagocytosis (1). Th2 cells are important for responses to helminthic and other gastrointestinal parasitic infections, producing IL-4, IL-5, and IL-10, and stimulating B cell differentiation (2). Th9 cells are a relatively newly defined subset, and are known to primarily produce IL-9 and facilitate the immune response against intestinal worms (3). Th17 cells are broadly involved in inflammation as well as host response to infection, producing IL-17, IL-6, and TNFα to recruit additional immune cell types to the site (4). In contrast with the pro-inflammatory nature of Teff cells, regulatory T (Treg) cells are responsible for immunosuppression, preventing overactive inflammatory responses and autoimmunity (5). This subset secretes key anti-inflammatory cytokines, notably TGFβ and IL-10, and Treg differentiation is driven by the transcription factor Foxp3. CD8^+^ cytotoxic T cells are primarily responsible for killing infected or malignant cells through the release of cytotoxic cytokines (TNFα, IFNγ) and granules (perforin, granzymes), and by initiating apoptotic processes mediated by the caspase cascade (6). Lastly, B cells are lymphocytes derived from bone marrow and are also major players within the adaptive immune system, supporting humoral immunity upon activation by producing large quantities of antibodies (7).

The metabolic profile of each of these specialized T cell subsets is optimized to support their unique functions (8). For example, activated CD4^+^ Teff cells, including Th1, Th2, Th17, and CD8^+^ cytotoxic T cells upregulate glucose uptake and glycolysis to promote rapid growth, proliferation, and effector function. Teff cells also rely on increased glutamine uptake and metabolism to support cell growth and proliferation, although the requirement for glutamine metabolism varies among T cell subsets (9). In contrast, Treg cells rely primarily on lipid oxidation to support their suppressive activity. While considerably less is known about B cell metabolism relative to T cell metabolism, there are some similarities such that naïve B cells are relatively quiescent, but following stimulation, have increased metabolic demand, likely to support proliferation and antibody production (10). Additionally, B cell subsets tend to display unique metabolic phenotypes as a product of their environment and function (11). Thus, the pathways that control adaptive immune cell function and metabolism are intimately linked (12–14). A number of recent advances indicate that immune cell identity, function, and metabolism are controlled, at least in part, via epigenetic mechanisms.

## Epigenetics

While DNA sequence is the same from cell to cell within an organism, the transcription (or lack thereof) of certain genes contributes greatly to the differentiation of the myriad of cell types that are present within an organism. This variation is controlled in large part by epigenetic mechanisms that result in dynamic but heritable changes in gene expression that do not involve changes in DNA sequence (15). There are several mechanisms by which this kind of transcriptome regulation may occur. One example of these mechanisms is DNA methylation, the addition of a methyl group to cysteine residues within DNA by various DNA methyltransferases typically in regions rich in cysteine-guanine dinucleotides. Generally, DNA methylation can act to either inhibit gene transcription (if methylation occurs within a promoter region) or promote transcription (if methylation occurs within the gene body) (16). Another mechanism of epigenetic regulation occurs via non-coding RNA. These single-stranded RNA fragments seek out complementary sites within the mRNA of target genes and degrade RNA, ultimately preventing translation (17). Lastly, the modification of histones is a highly prevalent mechanism of epigenetic control and contributes to genetic regulation by altering the physical structure of chromatin to improve or impair the accessibility of DNA to various transcription factors and transcription machinery. Histones form the backbone of the nucleosome, providing structure and stability. In contrast with the stability of DNA methylation, histone modifications can be more fluid over acute periods of time (18). Deacetylated histones form a densely packed chromatin structure, known as heterochromatin, physically preventing transcription. Histone acetylation maintains a more loose and fluid chromatin structure. Other common histone modifications can occur by methylation, phosphorylation, and deamination. Moreover, all of these mechanisms likely work synergistically to regulate the epigenome (19).

## Sirtuins

Among the four defined classes of histone deacetylases, sirtuins (class III) are a unique family of highly conserved, NAD^+^-dependent protein deacetylases with important implications on the epigenome. In addition to their deacetylase activity, sirtuins display some additional enzymatic function on other substrates, including ADP-ribosyltransferase and desuccinylase activity (20, 21). Mammalian sirtuins are orthologs of the silent information regulatory 2 (Sir2) protein, which was first identified in yeast as a significant contributor to the life-span extending effects of calorie restriction (22). These effects were further observed in *C. elegans* and *Drosophila* (23), suggesting that this pathway is conserved across species. Given the NAD^+^-dependent activity of sirtuins, they are activated in periods of catabolism and low nutrient availability, and were thus thought to be a novel target for mimicking the life-span extending effects of calorie restriction in humans.

Mammals ubiquitously express seven sirtuins (SIRT1-SIRT7) with different subcellular locations and functions. Sirtuins are currently gaining widespread attention in the context of a number of disease states associated with inflammation, including autoimmunity (24), cardiometabolic diseases (25), and cancers (26). Further, the mammalian sirtuins have been found to mediate cellular metabolism and adaptive responses to a variety of stresses, including calorie restriction and other metabolic stress (27). Given the relationship between nutrient availability and the function of the adaptive immune system (28), sirtuins are currently of great interest as mediators of tumor proliferation, autoimmunity, and the ability of an organism to respond to foreign pathogens.

**Figure 1:**
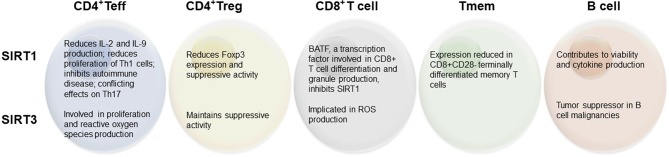
Summary of reported effects of SIRT1 and SIRT3 on lymphocyte populations.

The idea that sirtuins might be involved in immunity was posited over a decade ago (23), following the early finding that SIRT1 can regulate NF-κB (29), a transcription factor well-known to regulate inflammation and immune cell proliferation (30). Indeed, further study has begun to elucidate the link between this family of proteins and immune cell function. While each of the sirtuins has since been broadly studied, the most intense attention has been given to SIRT1 (primarily localized to the nucleus) and SIRT3 (primarily in mitochondria) with respect to adaptive immune cells, and will thus be the focus of this review (Figure 1). Further, considerations for the use of sirtuin-modifying drugs to manipulate immune activity are explored.

## Effector CD4^+^ T Cells

Activation of T cells occurs seconds after stimulation of the T cell antigen receptor (TCR) by a ligand, along with co-stimulatory signals, which initiate a number of signaling pathways that promote differentiation and growth. This activation is supported by a transition from a relatively quiescent oxidative metabolism to an intense glycolytic metabolic signature to support proliferation and cytokine production (12). This metabolic switch is likely driven, at least in part, by sirtuin activity, however there presently appears to be a number of incongruous effects reported across the various CD4^+^ T cell subsets.

SIRT1 appears to play a significant role in the regulation of Teff cell activation (31, 32). Early studies into the function of SIRT1 on Teff cells indicated that SIRT1 inhibits the immune response by acting as an antagonist against transcription factors that support IL-2 production (32) and thereby decreasing Th1 cell activation. This relationship may contribute in part to the link between fasting/calorie restriction and poor immune performance (28), given that SIRT1 is generally activated in response to fasting (33). The direct role of sirtuins within adaptive immune cells has also begun to be studied using knockout (KO) animal models. T cells from a SIRT1 KO mouse model are more proliferative, produce more IL-2 both *in vitro* and *in vivo*, and these mice are more susceptible to experimental autoimmune encephalomyelitis (EAE), indicating a more inflammatory immune phenotype (32). Studies in SIRT1 KO animals also found that T cells without SIRT1 can be activated solely via the T cell receptor (TCR), without co-stimulation by CD28, suggesting a hyper-sensitivity to activation signals when SIRT1 is not present (32). Follow-up studies indicated that IL-2 is involved in a feedback response to reduce further SIRT1 gene transcription and allow for proliferation in response to the activation cascade (34). Hyper-responsive Teff cells can contribute to an environment prone to autoimmune disease. In fact, earlier studies in SIRT1-null mice detailed the development of a mild autoimmune condition that resembled systemic lupus erythematosus, characterized by deposition of immune complexes within liver and kidneys, with some mice going on to spontaneously develop a diabetes insipidus-like autoimmune disorder after 2 years of age, altogether suggesting a preventative role of SIRT1 in autoimmunity (35).

SIRT1 also inhibits Bcl-2 Associated Transcription Factor 1 (Bclaf1) (36). Bclaf1 was originally identified as a promoter of apoptosis (37); however, subsequent studies revealed further reaching effects of Bclaf1 on T cell development, activation, and proliferation (38), perhaps by promoting hypoxia-inducible factor 1-α (HIF-1α) transcription (39). SIRT1-mediated inhibition of Bclaf1 is thought to occur by the binding of SIRT1 to the promoter region of Bclaf1 after stimulation of the TCR, suppressing acetylation of the histone 3 lysine 56 residue (H3K56) (36). When SIRT1 was knocked out of T cells, there was greater expression of the gene coding for Bclaf1, and specific knockdown of Bclaf was able to suppress the increase in IL-2 production and proliferation seen in SIRT1 KO mice (36).

SIRT1 inhibition can also depress the adaptive response and differentiation of Th2 cells. Pharmacological SIRT1 inhibition contributed to decreased allergic inflammation in BALB/c mice exposed to ovalbumin via aerosol (40). In addition, mice that exhibit KO of a transcriptional activator essential for Th2 differentiation (B-cell lymphoma/leukemia 11B; Bcl11b) have been found to be protected against EAE (41). Bcl11b is a transcriptional repressor and likely functions by recruiting SIRT1 for histone deacetylase activity (42). Although T helper 9 (Th9) cells exhibit a number of similarities to Th2 cells, SIRT1 inhibition has been found to promote Th9 cell differentiation and IL-9 production by these cells (43).

The implications of SIRT1 on Th17 cells have been more equivocal. Pharmacological induction of SIRT1 using resveratrol, low dose metformin, or the inhibitor SRT1720 has been shown to impair Th17 cell differentiation with decreased expression of IL-17 and RORγt, in a STAT3-dependent manner (44). The same study further describes anti-tumor effects of metformin by its action in reducing Th17 differentiation and STAT3 acetylation. In a separate study, *in vivo* activation of SIRT1 through treatment with NAD^+^ contributed to a delayed onset of EAE. This protection was hypothesized to be conferred by enhanced SIRT1 expression within the spinal cord of mice exposed to the EAE stimulus, which may suppress inflammatory responses by Th1 and Th17 cells (45). However, others have shown that SIRT1 is necessary for the production of pro-inflammatory Th17 cells through the deacetylation of transcription factor RORγt, which suggests that SIRT1 inhibitors could confer protection against autoimmunity (46). Clearly, more studies are needed to dissect out the role of SIRT1 in Th17 cell differentiation, proliferation, and cytokine response.

SIRT3 is a mitochondrial sirtuin that supports the structure, function, and biogenesis of the mitochondria (47). SIRT3 is elevated in fasting and calorie restriction in liver, muscle, and brown adipose tissue, and is known to modify cellular metabolism in those tissues (48, 49). In a model of experimental allogenic bone marrow transplantation, total T cells from donor animals that exhibit a whole-body SIRT3 KO were less likely to promote graft-vs.-host disease relative to T cells from control mice, but did not affect the graft-vs.-tumor effect, suggesting that targeted inhibition of SIRT3 in allogenic T cells can improve outcomes after transplant (50). Further, while SIRT3 KO did not affect the composition of peripheral naïve T cell subsets, it was determined that SIRT3 KO Teff cells were less proliferative and produced less reactive oxygen species (ROS) in response to non-specific TCR stimulation (50). However, a SIRT3 KO mouse model did not affect the development of immune cells or immune responses to various endotoxins (51), suggesting SIRT3 may play a limited role in Teff cell function.

Little is known about the role of the other sirtuins on Teff cell development and function. SIRT6 may be a negative regulator of glycolytic activity, notably through the inhibition of glucose transporter 1 (GLUT1) and the transcriptional regulator HIF-1α (52), suggesting a potential role for SIRT6 in downregulating Teff cell activation. HIF-1α is a transcriptional regulator of glycolysis and is known to regulate the production of a number of cytokines (53) and enhance Th17 cell differentiation (54). Relatively little work has been done on SIRT2 in adaptive immunity. However, SIRT2 has been identified as a potential suppressor of colitis through its deacetylase activity on NF-κB within bone marrow-derived macrophages in a mouse model (55). Further, this study observed a greater proportion of activated (CD4^+^CD69^+^) T cell populations at the mesenteric lymph nodes of SIRT2 KO mice in response to DSS-induced colitis, indicative of enhanced inflammatory action (55), and ascribing a role for SIRT2, similar to SIRT1, in limiting CD4^+^ Teff cell inflammation.

## Regulatory CD4^+^ T Cells

SIRT1 has been found to reduce the activity of Foxp3, contributing to an overall more inflammatory immune phenotype (46). Further, inhibiting SIRT1 can promote greater Treg suppressive activity (56, 57). While the regulation of Treg metabolism by sirtuins has not been widely studied, Treg function has been shown to be regulated in part by sirtuin activity. In addition, Foxp3 itself has been found to be a regulator of epigenetic activity to support the Treg phenotype, and is regulated in part by the deacetylase activity of SIRT1 (56). A previous review has outlined the role of demethylation and histone modifications that occur in order to promote and stabilize the expression of Foxp3 during Treg cell development (58). Briefly, three conserved non-coding sequences are primary targets for epigenetic mechanisms that regulate Foxp3 expression in response to external environmental stimulus.

Given the localization of SIRT3 to the mitochondria and its role in oxidative metabolism and mitochondrial function, it is not surprising that the loss of SIRT3 in Treg cells has been shown to impair their suppressive activity (59). Indeed, deletion of histone deacetylase 9 was found to be sufficient to increase Treg suppressive activity, by increasing the expression of SIRT3. Further, Treg cells from SIRT3 KO mice had impaired suppressive function both in an *in vitro* suppression assay and an *in vivo* cardiac allograft model, likely due to the role of SIRT3 in promoting oxidative metabolism (59).

## CD8^+^ T Cells

Activation and differentiation of CD8^+^ T cells leads to markedly variable chromatin accessibility (60), which is likely critical to facilitate the transition between naïve, effector, and memory CD8^+^ T cells. SIRT1 appears to play a crucial role in CD8^+^ T cell differentiation. Basic leucine zipper ATF-like transcription factor (BATF) has been shown to inhibit the expression of SIRT1, contributing to increased histone acetylation, particularly at the T-bet locus (61). This has been shown to affect CD8^+^ T cell differentiation and activity, as CD8^+^ T cells from BATF KO animals exhibited lower ATP production and lower mRNA expression of perforin and IFNγ (61). SIRT3 is also involved in CD8^+^ T cell function. Toubai et al. found that SIRT3-null activated CD8^+^ T cells produced less ROS upon activation (50). This impairment in ROS production may lead to impairments in sulfenylation, a process known to play a role in the regulation of histone deacetylases (62). Further SIRT3-null donor cells were able to attenuate graft-vs.-host disease within the gastrointestinal tract and the authors hypothesize this may be due, in part, to decreased CD8^+^ T cell trafficking to site (50).

## Memory T Cells

Following the primary immune response, a portion of T cells (CD4^+^ and CD8^+^) can become memory cells that remain ready to respond rapidly in the event that they re-encounter their antigen. These cells are relatively long-lived and therefore exhibit a relatively quiescent oxidative metabolism similar to that of a naïve immune cell until they are re-activated. Little is known about the role of sirtuins in mediating the generation or longevity of memory T cells; however, given the role of sirtuins in promoting oxidative metabolism, this is a potentially interesting area for further study. Though not specific to memory T cells, SIRT1 has distinct effects on PGC-1α and PGC-1β, both proteins with roles in facilitating mitochondrial biogenesis and oxidative metabolism. Thus, the return to a more oxidative metabolism in memory cells may be mediated in part by SIRT1, given the role of SIRT1-mediated deacetylation on transcription of PGC-1α and PGC-1β (63, 64). In support of this hypothesis, SIRT1 expression has been shown to be decreased in terminally differentiated CD8^+^CD28^−^ memory T cells, driving the downregulation of forkhead box protein O1 (FoxO1), a transcription factor that mediates T cell homing and differentiation (65). Further, these authors demonstrate that these SIRT1-low CD8^+^CD28^−^ memory cells have an enhanced glycolytic capacity in the resting state, which can support effector function upon reactivation.

## B Cells

Naïve B cells exhibit a relatively inert epigenetic status with high levels of DNA methylation and histone deacetylation. However, upon activation and maturation toward a germinal center B cell phenotype, B cells exhibit dramatic shifts in methylation status and become hypomethylated with increased histone acetylation and expression of various miRNAs (66, 67). Thus far, the limited literature on sirtuin activity in B cells indicates sirtuins support B cell viability, proliferation, and function. SIRT1 overexpression by viral transfection in BaF3 B cells (a murine B cell line) has been shown to support enhanced viability (mediated in part by a decrease in p53) and increased cytokine production (68). A short, non-coding microRNA, miR-132, is increased in B cells of patients with multiple sclerosis (MS) concurrent with a reduced expression of SIRT1 (69). SIRT3 also has been found to be a tumor suppressor in the context of B cell malignancies, as a number of malignant B cell lines display decreased SIRT3 protein expression and higher ROS levels, and overexpression of SIRT3 in these lines decreased proliferative activity (70). SIRT4 has also been shown to act as a tumor suppressor by inhibiting glutamine metabolism, which is necessary to conserve resources for repairing DNA damage (71). Further, SIRT4 overexpression can inhibit proliferation of Burkitt lymphoma cells, a model of B cell lymphoma (72). Inhibition of SIRT1 and SIRT2 increased apoptotic activity and ROS production in cells from patients with B cell chronic lymphotic leukemia (73). While not specific to sirtuins, histone deacetylase inhibitors have been shown to be effective in preventing B cell malignancies (74).

## Modulating Sirtuin Activity to Alter Immune Outcomes *in vivo*

A number of novel drugs with sirtuin modulatory activity have been studied in the context of immune function. However, it will be critical to determine whether to induce or inhibit sirtuin activity and, further, how to target specific sirtuins (perhaps even within a particular lymphocyte subset), in order to reach desired immune outcomes. For instance, promoting SIRT3 activity in Treg cells to improve suppressive capabilities and temper inflammation could be a novel means to treat autoimmunity. On the other hand, SIRT1 generally inhibits Teff inflammation, suggesting that activators of SIRT1 could be useful for the treatment of autoimmune disease; however, the effects of SIRT1 on T cells vary by subset and are context-dependent. As sirtuins are proteins with functions in a wide array of cell types, targeting specific sirtuins in specific tissues (and immune cell subsets) will remain an immense challenge.

Despite the challenges with tissue-specificity, drugs to modify sirtuin activity have been studied in cell culture and animal models. Inhibition of SIRT1 *in vivo* (using a SIRT1-specific inhibitor, EX-527) increased complications of sepsis at 12 h despite conferring dramatic protection at 24 h (75). EX-527 and sirtinol (another commonly studied sirtuin inhibitor with specificity against SIRT1 and SIRT2) also have been found to reduce platelet count (76). Metformin, classically prescribed as a medication for the management of type 2 diabetes, has been well-documented to have anticancer effects (77); however, the precise mechanism of action is largely unknown, though there is speculation that these effects may be due to the stimulatory effect of metformin on sirtuins. SIRT1 activation by metformin has been shown to decrease Th17 cell populations promoting a less inflammatory environment (44). SIRT1 knockdown has also been shown to promote apoptotic processes in leukemia cells (78), suggesting the exact mechanisms for sirtuin modulation in cancers is still being determined.

Resveratrol, a polyphenol compound with anti-inflammatory properties, is a compound with sirtuin modifying effects that is currently of intense interest within both the scientific and lay communities. Resveratrol increases SIRT1 activity and impedes acetylation of c-Jun, thereby limiting T cell activation (79). Further, resveratrol has been shown to improve outcomes in two well-characterized murine models of autoimmunity: EAE (80) and colitis (81). Additionally, resveratrol confers protection against a murine model of rheumatoid arthritis, by inhibiting Th17 expansion and IL-17 production, as well as autoantibody production from B cells (82). Resveratrol has also been shown to increase the ratio of CD4^+^ to CD8^+^ T cells and increase total Treg cells in the context of diet-induced obesity in mice while also conferring benefits on glucose homeostasis by activating phosphoinositide 3-kinase (PI3K) signaling pathways (83). Dosages necessary to produce these effects in humans are likely impossible to obtain exclusively through diet, but could realistically be obtained through supplementation.

The role of sirtuins in promoting organ transplant tolerance is also an area of intense investigation. While advances have been made in long-term survival following transplantation, current immunosuppressive therapies are known to promote infections and cancer. There is speculation that SIRT1 inhibitors may enhance the function of Treg cells to support immune suppression and allograft tolerance (84). Additionally, SIRT1 inhibitors may also confer prolonged allograft survival through the suppression of Th17 activity, as evidenced by decreased IL-17A (85); however, these results are in direct opposition to the SIRT1-activating and anti-tumor properties of metformin described above (44), and further studies are needed.

## Concluding Remarks

The wide-ranging effects of sirtuins and the availability of a number of sirtuin-modifying compounds provide a significant opportunity for future study to improve immune cell phenotypes. However, there are significant challenges ahead in developing drugs with targeted tissue-specific effects given the ubiquity of these mechanisms within the body. The development of tissue-specific and sirtuin-specific therapies remains an intriguing possibility to treat the myriad of autoimmune diseases, cancers, and other chronic diseases associated with inflammation that are now understood to be regulated by some degree of protein acetylation.

## Author Contributions

All authors listed have made a substantial, direct and intellectual contribution to the work, and approved it for publication.

### Conflict of Interest Statement

The authors declare that the research was conducted in the absence of any commercial or financial relationships that could be construed as a potential conflict of interest.
